# Mouse-derived Synaptosomes Trypsin Cleavage Assay to Characterize Synaptic Protein Sub-localization

**DOI:** 10.21769/BioProtoc.5164

**Published:** 2025-01-20

**Authors:** Jasmeet Kaur Shergill, Domenico Azarnia Tehran

**Affiliations:** 1 Department of Nanophysiology, Rheinland-Pfälzische Technische Universität Kaiserslautern-Landau (RPTU), Kaiserslautern, Germany; 2 Department of Structural Interactomics, Leibniz-Forschungsinstitut für Molekulare Pharmakologie (FMP), Berlin, Germany

**Keywords:** Synaptosomes, Trypsin, Fractionation, Synapse, Presynaptic, Postsynaptic, Neurotransmission, Western blotting

## Abstract

Neurons communicate through neurotransmission at highly specialized junctions called synapses. Each neuron forms numerous synaptic connections, consisting of presynaptic and postsynaptic terminals. Upon the arrival of an action potential, neurotransmitters are released from the presynaptic site and diffuse across the synaptic cleft to bind specialized receptors at the postsynaptic terminal. This process is tightly regulated by several proteins at both presynaptic and postsynaptic sites. The localization, abundance, and function of these proteins are essential for productive neurotransmission and are often affected in neurological and neurodegenerative disorders. Here, we outline a method for purifying mouse synaptosomes and using limited tryptic digestion to assess the subcellular localization of synaptic proteins. During synaptosomes purification, presynaptic terminals reseal and are protected from proteolysis, while postsynaptic proteins remain susceptible to tryptic cleavage. These changes can easily be evaluated by western blot analysis. This approach offers a straightforward and reliable method to evaluate the subcellular localization of synaptic proteins based on their proteolytic sensitivity, providing valuable insights into synaptic physiology and pathology.

Key features

• Builds upon the method developed by Boyken et al. [1] and introduces the use of isolated mouse synaptosomes to assess synaptic protein sub-localization.

• Limited tryptic digestion differentiates between presynaptic and postsynaptic proteins based on proteolytic sensitivity.

• Requires standard biochemical reagents and western blotting equipment and can be completed in two/three days, including synaptosome purification and western blot analysis.

Graphical overview

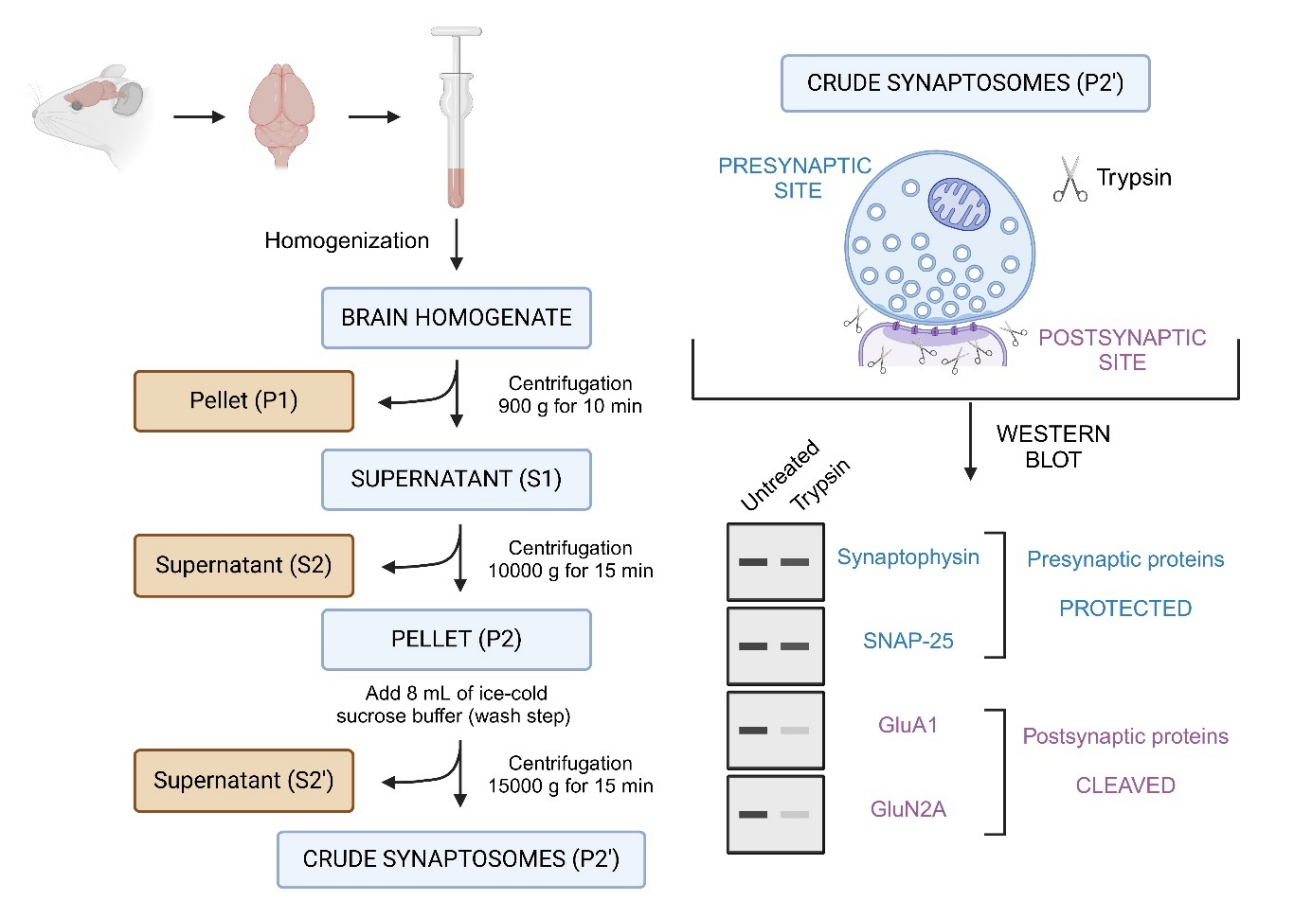

**Overview of the synaptosomes trypsin cleavage assay.** This protocol describes the isolation of synaptosomes from mouse brain tissue, followed by limited trypsin digestion to assess the compartmental localization of synaptic proteins. Synaptosomes, which are isolated via differential centrifugation, consist of resealed presynaptic terminals that are protected from proteolysis, while the exposed postsynaptic compartments are accessible to trypsin and undergo proteolytic cleavage. Following digestion, samples are analyzed using SDS-PAGE and western blotting. Proteins of interest are probed using specific antibodies to determine whether they are presynaptic or postsynaptic. Presynaptic proteins (e.g., synaptophysin, SNAP-25) remain intact, while postsynaptic proteins (e.g., GluA1, GluN2A) are cleaved.

## Background

Neurotransmission is the fundamental process by which neurons communicate and relay information, enabling neuronal circuits to function effectively, thus governing higher-order functions such as memory formation and storage [2]. This communication occurs at specialized structures called synapses, which consist of presynaptic and postsynaptic terminals [2–5]. In the presynaptic neuron, upon the arrival of an action potential, neurotransmitter-containing synaptic vesicles fuse with the presynaptic membrane, releasing their contents into the synaptic cleft [2–5]. These neurotransmitters diffuse across the cleft and bind to specialized receptors at the postsynaptic terminal, triggering the transmission of signals in the postsynaptic neuron [2–5]. This entire process is tightly regulated by a complex set of proteins [6–7]. Understanding the localization, abundance, and function of these proteins is essential for uncovering the mechanisms that regulate synaptic transmission, which can be altered in a variety of neurological and neurodegenerative disorders, such as Alzheimer's disease, Parkinson’s disease, and schizophrenia [8–9].

Several methodologies have been developed to study the protein composition of synapses [10]. Techniques such as immunohistochemistry and mass spectrometry have been widely used to map synaptic proteins [11–14]. Although these methods offer valuable insights into synaptic protein localization and abundance, they lack the resolution to distinguish between presynaptic and postsynaptic sites, which are separated by the synaptic cleft (approximately 20 nm wide). More recently, super-resolution microscopy has offered this level of resolution, but it often involves significant sample preparation time and is less suitable for high-throughput analysis [15–16]. To address some of these limitations and to isolate synaptic vesicle docking complexes, a method involving the use of synaptosomes combined with limited tryptic digestion has been developed by Boyken et al. [1]. This approach allows for the study of synaptic proteins in a more high-throughput and compartment-specific manner. Synaptosomes are partially resealed structures derived from isolated nerve terminals from mouse brains, in which presynaptic terminals remain sealed and intact while the postsynaptic terminal is left unprotected. By applying limited tryptic digestion to these synaptosomes, presynaptic proteins are protected from proteolysis, as they remain inaccessible to trypsin, while postsynaptic proteins are vulnerable to cleavage [1]. This method thus enables the distinction between presynaptic and postsynaptic proteins by western blot analysis, offering an efficient way to assess their localization.

To our knowledge, the protocol presented here has been used to study the molecular architecture of active zones [1], to confirm the postsynaptic localization of Synaptotagmin-3 [17], to assess the large postsynaptic pools of CALM [18] and intersectin1 (ITSN1) [19], and to confirm the postsynaptic localization of the total and phosphorylation forms of p85S6K over p70S6K, which instead appear to be mainly presynaptic [20]. Nevertheless, this protocol has the potential to be applied in various contexts in combination with mass spectrometry to enable a more precise understanding of the molecular components of synapses.

## Materials and reagents

**Biological materials**

1. Mouse (*Mus musculus*, C57BL/6J) brain (preferably P60 or above)

**Reagents**

1. Sucrose (ROTH, catalog number: 9097.1)

2. HEPES (ROTH, catalog number: 6763.2)

3. Trypsin (Sigma-Aldrich, catalog number: T1005)

4. Bradford reagent (Sigma-Aldrich, catalog number: B6916)

5. Tris (ROTH, catalog number: AE15.6)

6. Glycerin (ROTH, catalog number: 3783.3)

7. SDS (ROTH, catalog number: 2326.2)

8. 2-Mercaptoethanol (Sigma Aldrich, catalog number: M3148)

9. Bromophenol blue (ROTH, catalog number: T116.1)

10. HCl (ROTH, catalog number: 4625.1)

11. Glycine (ROTH, catalog number: 3790.3)

12. Methanol (ROTH, catalog number: 8388.6)

13. Ponceau S (ROTH, catalog number: 5938.1)

14. Acetic acid (ROTH, catalog number: 3738.2)

15. Disodium hydrogen phosphate (Na_2_HPO_4_) (ROTH, catalog number: T876.1)

16. Potassium dihydrogen phosphate (KH_2_PO_4_) (ROTH, catalog number: 3904.1)

17. Sodium chloride (NaCl) (ROTH, catalog number: 3957.1)

18. Potassium chloride (KCl) (ROTH, catalog number: 6781.1)

19. Tween-20 (Polysorbat) (Fischer Scientific, catalog number: T/4206/60)

20. Intercept blocking buffer (LI-COR, catalog number: 927-60001)

21. ROTIPHORSE Gel 30 (37.5:1), ready-to-use acrylamide (ROTH, catalog number: 3029.1)

22. Ammonium peroxydisulfate (APS) (ROTH, catalog number: 9592.5)

23. TEMED (ROTH, catalog number: 2367.1)

24. Bovine serum albumin fraction V (BSA) (Sigma-Aldrich, catalog number: 1.12018.0100)

25. 2-propanol (isopropanol) (VWR, catalog number: 20842.323)

26. PageRuler prestained protein ladder (Thermo Scientific, catalog number: 26616)

27. Blotting paper (MACHEREY-NAGEL, catalog number: MN 827 B)

28. Nitrocellulose membrane (Amersham, catalog number: 10600004)

29. Anti-GluA1 antibody (Merk Millipore, catalog number: MAB2263)

30. Anti-GluA2 antibody (Merk Millipore, catalog number: MAB397)

31. Anti-GluN2A antibody (Cell signalling, catalog number: #4205)

32. Anti-GluN2B antibody (Cell signalling, catalog number: #4207)

33. Anti-Homer1 antibody (Synaptic Systems, catalog number: 160003)

34. Anti-Synaptotagmin1 antibody (SYT1) (Synaptic Systems, catalog number: 105103)

35. Anti-Synaptophysin1 antibody (SYP1) (Synaptic Systems, catalog number: 101011)

36. Anti-Snap25 antibody (Synaptic Systems, catalog number: 111011)

37. IRDye 800CW goat anti-Rabbit IgG secondary antibody (LI-COR, catalog number: 926-32211)

38. IRDye 680RD goat anti-Mouse IgG secondary antibody (LI-COR, catalog number: 926-68070)

**Solutions**

1. Sucrose buffer (see Recipes)

2. BSA solution for Bradford assay (see Recipes)

3. Trypsin solution (see Recipes)

4. 6× Loading sample buffer (LSB) (see Recipes)

5. 4× separating gel buffer (see Recipes)

6. 4× stacking gel buffer (see Recipes)

7. 10× running buffer (see Recipes)

8. 1× running buffer (see Recipes)

9. 10× transfer buffer (see Recipes)

10. 1× transfer buffer (see Recipes)

11. Ponceau S staining solution (see Recipes)

12. 10× PBS (see Recipes)

13. 1× PBS (see Recipes)

14. 1× PBST (see Recipes)

15. Blocking solution (see Recipes)

16. 10% APS solution (see Recipes)

**Recipes**

**1. Sucrose buffer**

**Table:** 

Reagent	Final concentration	Quantity or Volume
Sucrose	0.32 M	10.95 g
HEPES	5 mM	0.119 g
Water	n/a	100 mL
Total	n/a	100 mL

Adjust the pH to 8.0 before reaching the final volume.

**2. BSA stock solution for Bradford assay**

**Table:** 

Reagent	Final concentration	Quantity or Volume
BSA	2 mg/mL	2 mg
Sucrose buffer	n/a	1 mL
Total	n/a	1 mL

**3. Trypsin solution**

**Table:** 

Reagent	Final concentration	Quantity or Volume
Trypsin	0.1 mg/mL	1 mg
Water	n/a	10 mL
Total	n/a	10 mL

Store the solution on ice after preparation.

**4. 6× loading sample buffer (LSB)**

**Table:** 

Reagent	Final concentration	Quantity or Volume
Tris pH 6.8	375 mM	2.268 g
Glycerin	50%	25 mL
2-mercaptoethanol	10%	5 mL
SDS	10%	5 g
Bromophenol blue	0.03%	15 mg
Total	n/a	50 mL

Heat the solution to 55 °C to dissolve the SDS, then add 2-mercaptoethanol at the end.

**5. 4× Separating gel buffer**

**Table:** 

Reagent	Final concentration	Quantity or Volume
Tris pH 8.8	1.5 M	90.86 g
SDS	0.4%	2 g
Water	n/a	500 mL
Total	n/a	500 mL

Tris base pH level is adjusted to 8.8 using HCl. Add SDS at the end.

**6. 4× Stacking gel buffer**

**Table:** 

Reagent	Final concentration	Quantity or Volume
Tris pH 6.8	0.5 M	30.29 g
SDS	0.4%	1 g
Water	n/a	250 mL
Total	n/a	250 mL

Tris base pH level is adjusted to 6.8 using HCl. Add SDS at the end.

**7. 10× Running buffer**

**Table:** 

Reagent	Final concentration	Quantity or Volume
Tris pH 8.4	250 mM	30.29 g
Glycine	1.92 M	144.13 g
SDS	1%	10 g
Water	n/a	1,000 mL
Total	n/a	1,000 mL

**8. 1× Running buffer**

**Table:** 

Reagent	Final concentration	Quantity or Volume
10× Running buffer	1×	100 mL
Water	n/a	900 mL
Total	n/a	1,000 mL

**9. 10× Transfer buffer**

**Table:** 

Reagent	Final concentration	Quantity or Volume
Tris	250 mM	30.29 g
Glycine	1.92 M	144.13 g
Water	n/a	1,000 mL
Total	n/a	1,000 mL

**10. 1× Transfer buffer**

**Table:** 

Reagent	Final concentration	Quantity or Volume
10× Transfer buffer	1×	100 mL
Methanol	20%	200 mL
Water	n/a	700 mL
Total	n/a	1,000 mL

**11. Ponceau S staining solution**

**Table:** 

Reagent	Final concentration	Quantity or Volume
Ponceau S	0.5%	1.25 g
Acetic acid	1%	2.5 mL
Water	n/a	250 mL
Total	n/a	250 mL

**12. 10× PBS**

**Table:** 

Reagent	Final concentration	Quantity or Volume
Na_2_HPO_4_	100 mM	11.49 g
KH_2_PO_4_	18 mM	2.45 g
NaCl	1.4 M	82.82 g
KCl	27 mM	2.01 g
Water	n/a	1,000 mL
Total	n/a	1,000 mL

**13. 1× PBS**

**Table:** 

Reagent	Final concentration	Quantity or Volume
10× PBS	1×	100 mL
Water	n/a	900 mL
Total	n/a	1,000 mL

**14. 1× PBST**

**Table:** 

Reagent	Final concentration	Quantity or Volume
10× PBS	1×	100 mL
Tween-20	0.1%	1 mL
Water	n/a	900 mL
Total	n/a	1,000 mL

**15. Blocking solution**

**Table:** 

Reagent	Final concentration	Quantity or Volume
LiCOR blocking solution	50%	50 mL
PBST	50%	50 mL
Total	n/a	100 mL

**16. 10% APS solution**

**Table:** 

Reagent	Final concentration	Quantity or Volume
APS	10%	1 g
Water	n/a	10 mL
Total	n/a	10 mL

**Laboratory supplies**

1. Falcon 15 mL centrifuge tube (CORNING, catalog number: 352096)

2. Falcon 50 mL centrifuge tube (CORNING, catalog number: 352070)

3. Ultra-high performance centrifuge tubes (VWR, 525-1085)

4. SurPhob tips EcoReload, 1,250 μL (Biozym, catalog number: VT0174)

5. SurPhob tips EcoReload, 200 μL (Biozym, catalog number: VT0144)

6. SurPhob tips EcoReload, 10 μL (Biozym, catalog number: VT0104)

7. Transferpette S pipette 100–1,000 μL (BRAND, catalog number: BR705880)

8. Transferpette S pipette 20–200 μL (BRAND, catalog number: BR705878)

9. Transferpette S pipette 0.1–2.5 μL (BRAND, catalog number: BR705869)

10. Reaction tubes 1.5 mL (Biozym, catalog number: 710310)

11. 96-well flat bottom plate (Anicrin, catalog number: M09600P0)

12. Ice buckets for cooling steps

## Equipment

1. Water bath (Lauda, model: Hydro H 20 S)

2. Centrifuge (Eppendorf, model: 5910 Ri, rotor: FA-6x50)

3. Odyssey Fc imaging system (Li-COR Odyssey Fc, model: 2800)

4. Homogenizer (Heidolf, model: Hei-TORQUE core)

5. Tissue grind pestle (Kimble Chase, catalog number: 885481-0023)

6. Mini-Protean Tetra cell casting modules (Bio-Rad, catalog number: 1658050)

7. Mini-Protean Tetra cell 4-gel system (Bio-Rad, catalog number: 1658004)

8. Criterion Blotter with wire electrodes (Bio-Rad, catalog number: 1704071)

9. Thermal shaker (VWR, model: Thermal shake lite, catalog number: 460-0249P)

10. SpectroStar Nano V5.70 (BMG Labtech)

## Software and datasets

1. Image studio (Li-COR, Version 5.2)
2. MARS data analysis software (BMG Labtech, Version 3.42)

## Procedure

**A. Crude synaptosome preparation**

*Note: Ensure to maintain ice-cold conditions throughout all steps.*

1. Sacrifice two mice by cervical dislocation and immediately dissect the brains.

*Note: Treat each brain separately in different tubes for the following steps.*

2. Place the brain in a pre-chilled tissue grinding pestle and add 8 mL of ice-cold sucrose buffer.

3. Homogenize at 900 rpm using 12 up-and-down strokes with the homogenizer.

4. Transfer the homogenate into a 15 mL centrifuge tube.

5. Spin the homogenate at 900× *g* for 10 min at 4 °C to remove large debris.

6. Transfer the supernatant (S1) into a 15 mL centrifuge tube and discard the pellet (P1).

7. Spin the collected supernatant (S1) in a centrifuge at 10,000× *g* for 15 min at 4 °C.

8. Discard the supernatant (S2) and resuspend the pellet (P2) in 2 mL of ice-cold sucrose buffer by gently pipetting up and down 3–4 times.

9. Add 6 mL of ice-cold sucrose buffer and gently invert to mix.

10. Spin the sample at 15,000× *g* for 15 min at 4 °C.

11. Discard the supernatant (S2’) and resuspend the pellet (P2’: crude synaptosomes) in 2 mL of sucrose buffer (see graphical overview for a schematic representation of synaptosomes preparation).

12. Combine the crude synaptosomes obtained from two brains, 4 mL in total, in a single 15 mL tube.

**B. Protein estimation (Bradford assay)**

1. Mix the Bradford reagent well before use and allow it to come to room temperature.

2. Prepare protein standards using a stock solution of 2 mg/mL BSA in sucrose buffer as mentioned in Table 1.

**Table 1.** Dilution scheme for protein standards using 2 mg/mL of BSA in sucrose buffer.

Vial	Sucrose buffer	Volume and source of BSA	Final BSA concentration
A	0	300 μL of stock	2 mg/mL
B	125 μL	370 μL of stock	1.5 mg/mL
C	325 μL	325 μL of stock	1 mg/mL
D	175 μL	175 μL of vial B dilution	0.75 mg/mL
E	325 μL	325 μL of vial C dilution	0.5 mg/mL
F	325 μL	325 μL of vial E dilution	0.25 mg/mL
G	400 μL	0	0 mg/mL (blank)

3. Add 200 μL of Bradford reagent to a 96-well plate, add 2 μL of your protein standards or your sample (crude synaptosomes) to the reagent, and mix.

*Note: Perform each measurement in triplicate and ensure that your actual sample concentration falls within the linear range of your standard curve, as other concentrations may lead to a nonlinear response.*

4. Incubate for 5 min at room temperature in the dark.

5. Measure absorbance at 595 nm using the SpectroStar Nano V5.70.

6. Determine protein concentration using the MARS data analysis software.

*Note: The usual protein concentration obtained from a single P60 mouse brain is 2–3 mg/mL (if the synaptosome yield is lower than expected, please refer to the troubleshooting section).*

**C. Proteolytic digestion procedure**

1. For the untreated sample, add 5 mg of crude synaptosomes in a final volume of 10 mL of sucrose buffer without adding trypsin.

2. To initiate proteolytic digestion and to reach a final protein–protease ratio of 100:1 (see troubleshooting section), add 500 µL of trypsin solution (0.1 mg/mL) to 5 mg of crude synaptosomes in a final volume of 10 mL of sucrose buffer (see troubleshooting section).

3. Incubate the untreated sample and the protein–protease mixture for 10 min at 30 °C in a water bath, with occasional gentle inversion (no vortexing).

4. Centrifuge the samples at 8,700× *g* for 3 min at 4 °C.

5. Quickly resuspend the pellets in 1 mL of sucrose buffer containing 200 μL of 6× loading sample buffer to stop protease activity and in order to reach a final concentration of 5 µg/µL.

6. Boil the samples at 95 °C for 10 min.

7. The samples are now ready for SDS-PAGE to determine the subcellular localization of your protein of interest.

*Pause point: Samples can be stored at -20 °C, and the following steps can be performed the day after. In our hands, samples can be stored at -20 °C for three months without degradation.*

**D. SDS gel casting and running**

1. Clean the glass plates thoroughly and insert them into the casting frame.

2. Prepare the 10% separating gel solution (Table 2) in a 15 mL tube and swirl the solution gently.

**Table 2.** Recipe for 10% separating gel

Reagent	Quantity or Volume
Water	3 mL
4× separating buffer	1.875 mL
Acrylamide/bis-acrylamide	2.5 mL
10% APS solution	150 μL
TEMED	15 μL

3. Pipette the separating gel solution between the glass plates, leaving 2 cm at the top.

4. Overlay the separating gel with isopropanol, making the separating gel regular and linear.

5. Once set, pour off completely the isopropanol and let it dry for 5 min.

6. Prepare the stacking gel solution (Table 3) in a 15 mL tube and swirl the solution gently.

**Table 3.** Recipe for 3.8% stacking gel

Reagent	Quantity or Volume
Water	1.625 mL
4× stacking buffer	0.625 mL
Acrylamide/bis-acrylamide	0.333 mL
10% APS solution	75 μL
TEMED	7.5 μL

7. Pipette the stacking gel solution into the gap between the glass plates, layering it on top of the set separating gel.

8. Quickly insert the comb, ensuring to avoid any air bubbles, and allow it to sit for 10 min to set.

9. Carefully remove the comb once the stacking gel is fully set.

10. Take the gel out of the casting stand and insert it into the running cell buffer dam.

11. Pour 1× running buffer into the inner and outer chambers of the gel running apparatus and remove the comb.

12. Load the untreated and trypsin-treated samples (20–30 μg) into the wells along with a protein ladder/marker for size reference.

13. Run the gel at 80 V for the staking phase (approximately 40 min) and then increase to 130 V for the resolving phase, running until the dye front reaches the bottom of the gel.

**E. Western blotting**

1. Prepare the transfer materials by soaking the sponge, blotting paper, and nitrocellulose (NC) membrane in cold 1× transfer buffer.

2. Assemble the sandwich for the transfer in the following order: sponge—blotting paper—NC membrane—blotting paper—sponge.

3. Use a roller to gently remove any air bubbles between the layers for even transfer.

4. Fill the transfer chamber with cold 1× transfer buffer and insert the cool pack to maintain the temperature.

5. Insert the sandwich into the transfer chamber and run the transfer at 110 V for 100 min in a cold room or with the chamber on ice to prevent overheating.

**F. Membrane staining**

**Figure 1.**
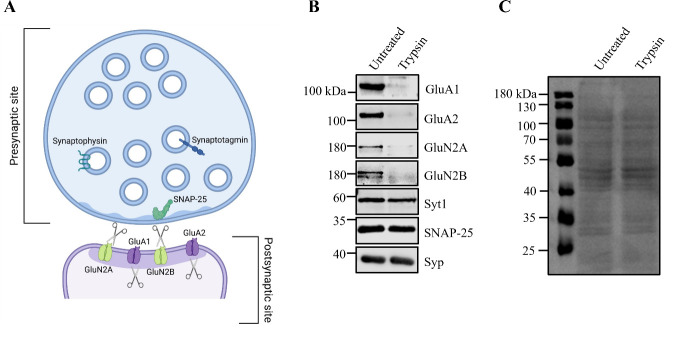
Tryptic digest of synaptosomes reveals subcellular localization of synaptic proteins. A. Schematic representation of synaptosomes showing the presynaptic terminal resealed into an enclosed compartment, protecting presynaptic proteins from trypsin proteolysis, while postsynaptic proteins remain susceptible to tryptic digestion. B. Synaptosomes are either left untreated or incubated with trypsin, followed by immunoblot analysis. Presynaptic proteins, such as synaptotagmin1 (Syt1), SNAP-25, and synaptophysin1 (Syp), are protected from proteolysis. In contrast, postsynaptic proteins, including the AMPAR subunits GluA1 and GluA2, as well as the NMDAR subunits GluN2A and GluN2B, are sensitive to tryptic digestion, indicating their postsynaptic localization. C. Example of a Ponceau S staining of synaptosomes, either untreated or incubated with trypsin for 10 min at 30 °C. Please note that in the trypsin-treated sample, only postsynaptic proteins are cleaved. Therefore, there should not be a significant difference from the untreated sample. If many bands disappear, this may indicate over-digestion (see troubleshooting section).

1. Carefully remove the NC membrane after the transfer and immediately stain it with Ponceau S staining solution for a few minutes to visualize the protein bands.

2. Rinse the membrane with water and take an image to record the transferred protein bands (see Figure 1).

3. Wash the membrane several times with 1× PBST until all traces of Ponceau S stain are gone.

4. Block the membrane by incubating it in blocking solution for 30–60 min to prevent nonspecific antibody binding.

5. While the membrane is blocking, prepare the primary antibody solution in a 15 mL tube using the blocking solution at the required dilution (see Table 4).

**Table 4.** Antibodies dilutions used in Figure 1

Antibody	Dilution used	Source
GluR1	1:1,000	Mouse
GluR2	1:1,000	Mouse
GluN2A	1:200	Rabbit
GluN2B	1:200	Rabbit
Synaptotagmin1	1:500	Rabbit
Synaptophysin1	1:500	Mouse
SNAP-25	1:500	Mouse

6. Transfer the membrane into the tube containing the primary antibody solution and incubate overnight at 4 °C on a rotator to ensure uniform binding.

7. The following day, wash the membrane with 1× PBST three times for 10 min each to remove any unbound primary antibodies.

8. Prepare the secondary antibody (mouse or rabbit, depending on the primary antibody used) solution at the dilution of 1:10,000 in 1× PBST.

9. Incubate the membrane with the secondary antibody for 45 min to 1 h at room temperature.

10. Wash the membrane with 1× PBST three times for 10 min each and then twice with 1× PBS to remove unbound secondary antibodies.

11. Visualize the membrane using the LiCOR imaging system to detect the protein of interest (see Figure 1) (refer to the troubleshooting section if variability in the proteolytic cleavage of postsynaptic proteins, unexpected degradation, or digestion of presynaptic proteins is observed).

## Data analysis

The protocol essentially follows the workflow of the original synaptosome cleavage assay developed by Boyken et al. [1]. For data analysis in our publications [18–19], bands corresponding to synaptic proteins were quantified using the Empiria Studio Software package (LI-COR Biosciences). Band intensities of trypsin-treated samples were normalized to the untreated control, which is set to 100%, to calculate the relative abundance of each protein. A minimum of three biological replicates (n = 3) are recommended to ensure reproducibility, with data plotted as mean ± SEM. Statistical comparisons between groups (treated vs. untreated samples) can be performed using a one-sample t-test, comparing the relative abundance of trypsin-treated proteins to the hypothetical value of 100% from the untreated control. A p-value < 0.05 is considered significant. Examples of data analysis, quantification, and representation can be found in Figure 1B–C from [18] and Figure 4A from [19].

## Validation of protocol

**Figure 2.**
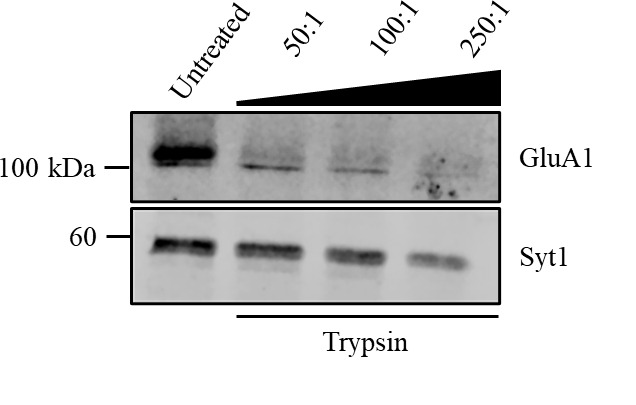
Determination of optimal protein-to-protease ratio for the trypsin cleavage assay. Representative immunoblot showing the effects of varying protein-to-protease ratios (50:1, 100:1, and 250:1) on the cleavage of the postsynaptic protein GluA1 and the presynaptic protein synaptotagmin1 (Syt1). Synaptosomes were left untreated or were treated with increasing trypsin concentrations. GluA1 is sensitive to trypsin digestion across all conditions. In contrast, Syt1 is resistant at the 50:1 ratio but becomes partially cleaved at higher trypsin concentrations, demonstrating the importance of optimizing the trypsin concentration to preserve presynaptic proteins.

To our knowledge, this protocol or parts of it has been used and validated in the following research articles:

- Boyken et al. [1]. Molecular Profiling of Synaptic Vesicle Docking Sites Reveals Novel Proteins but Few Differences between Glutamatergic and GABAergic Synapses. *Neuron* (Figure 1, panel B; Figure 2, panel A).
  The protocol was originally developed for the isolation of a fraction highly enriched in synaptic vesicles docked to active zone. Mild proteolysis of synaptosomes was used to dissociate the presynaptic from the postsynaptic membrane.
- Awasthi et al. [17]. Synaptotagmin-3 drives AMPA receptor endocytosis, depression of synapse strength, and forgetting. *Science* (Figure 1, panel E and F).
  The protocol was used to verify the subcellular localization of synaptotagmin-3 (Syt-3). Awasthi et al. demonstrated that presynaptic proteins (such as synapsin, synaptobrevin-2, and Rab3a) were protected from trypsin cleavage, while postsynaptic proteins (such as Homer, GluA1, and PSD95), including Syt3, were cleaved.
- Azarnia Tehran et al. [18]. Selective endocytosis of Ca^2+^-permeable AMPARs by the Alzheimer’s disease risk factor CALM bidirectionally controls synaptic plasticity. *Science Advances* (Figure 1, panel B and C).
  The protocol was used to reveal the subcellular localization of the endocytic adaptor clathrin assembly lymphoid myeloid leukemia protein (CALM). We consistently found that while presynaptic proteins (such as SNAP-25, synaptophysin, and Rab3a) were protected from proteolysis, CALM was sensitive to trypsin, indicative of a large postsynaptic pool.
- Li et al. [20]. P85S6K sustains synaptic GluA1 to ameliorate cognitive deficits in Alzheimer’s disease. *Translational Neurodegeneration* (Figure 1, panel C).
  The protocol was used to verify the subcellular localization of p85S6K. As expected, presynaptic proteins (such as synaptophysin) were protected, while postsynaptic proteins (such as GluA1 and PSD95) were cleaved. Li et al. found that p85S6K, in both total and phosphorylated forms, was also sensitive to tryptic digestion, revealing its postsynaptic localization.
- Vollweiter et al. [19]. Intersectin deficiency impairs cortico-striatal neurotransmission and causes obsessive–compulsive behaviors in mice. *Proc Natl Acad Sci USA* (Figure 4, panel A).
  The protocol was used to verify the subcellular localization of intersectin1 (ITSN1). We found that ITSN1 is equally distributed between presynaptic and postsynaptic sites.

## General notes and troubleshooting

**General notes**

1. This protocol requires basic knowledge of laboratory techniques and minimal animal surgical procedures.

2. To ensure optimal preservation of protein integrity during synaptosome isolation, it is critical that all procedures involving the preparation of synaptosomes are carried out on ice or at 4 °C. Always pre-chill your reagents and equipment (e.g., centrifuges) before starting the experiment.

3. In our publications, we used C57BL/6J mice, both male and female, above P60, at which point synaptogenesis and synaptic pruning are largely completed, and synaptic architecture is fully developed. The protocol should work for other strains and genetic backgrounds, with minimal adjustments. If the goal of the experiment is to assess developmental changes, using younger mice could provide valuable insights. However, some adjustments are recommended. For instance, approximately 3–4 times the number of brains may be needed to achieve similar protein yields. The same considerations apply to synaptosomes derived from specific mouse brain regions, which may be relevant in neurodegenerative diseases where only particular brain regions are affected, or from cultured neurons. Exact scaling should be determined experimentally when tissue availability is limited. For example, preliminary testing with different volumes is recommended to ensure effective synaptosome isolation.

4. The material obtained from two mouse brains is sufficient for both undigested and digested samples and can be used to check several known presynaptic and postsynaptic proteins as control, along with the protein(s) of interest via western blot. Since 5 mg of crude synaptosomes is enough for one experimental point, using two mouse brains also provides enough material to identify the optimal trypsin concentration (see troubleshooting section). However, if the optimal trypsin concentration is already known and fewer proteins need to be analyzed, the experiment can be scaled down to use a single mouse brain. In any case, we recommend verifying synaptosome integrity and confirming experimental success by checking at least a couple of known presynaptic and postsynaptic markers as control.

5. Due to trypsin digestion, we often observe bands at a lower molecular weight than the full-length postsynaptic protein of interest. This occurs because the antibody may also recognize peptide fragments generated by trypsin cleavage.

6. If proteins are partially cleaved (50%–70% remains), this may indicate that the protein is present at both presynaptic and postsynaptic ends, as seen with CALM and intersectin1 [18–19]. Nonetheless, we recommend combining results from the synaptosome trypsin cleavage assay with STED microscopy staining, using both presynaptic and postsynaptic markers. The combination of these methodologies will increase confidence in determining the sub-localization of your protein(s) of interest.

7. This protocol has been routinely used in our labs to assess the presynaptic and postsynaptic localization of various proteins by western blotting. However, it also has potential applications in combination with mass spectrometry. For example, the supernatant of trypsinized synaptosomes can be precipitated using chloroform–methanol precipitation, followed by further digestion with LysC and trypsin overnight at 37 °C, and then analyzed via mass spectrometry to identify postsynaptic proteins in high-throughput manner. Similarly, the pellet from trypsinized synaptosomes can be quickly lysed in lysis buffer (100mM Tris, 1% sodium deoxycholate, 10mM TCEP, and 12mM 2-chloroacetamide) by repeated sonication, followed by digestion with LysC and trypsin overnight at 37 °C to identify presynaptic proteins. In both cases, the resulting dry peptides can be stored at -80 °C.

8. Based on our experience, postsynaptic proteins (e.g., GluA1) show an optimal cleavage range of 90%, with an acceptable range of 80%–90%, meaning that the remaining protein after trypsin treatment should be 0%–20%. In contrast, presynaptic proteins (e.g., Syt1) have an optimal cleavage range of 0%–10%, with an acceptable extended range of 10%–20%, so that the remaining protein amount should be 80%–100%. When proteins show intermediate cleavage levels (40%–70%), these proteins are inferred to localize to both presynaptic and postsynaptic sites.

**Troubleshooting**

Problem 1: Low yield of synaptosomes.

Possible cause: Low yield of synaptosomes can result from insufficient tissue homogenization, improper centrifugation, or poor buffer quality.

Solution: We recommend using a mechanical homogenizer, as it provides the consistent shear force necessary for effective brain disruption while preserving synaptic structures. However, we noticed that other published protocols have successfully used a Dounce homogenizer, with 15–20 strokes using a loose pestle to achieve comparable disruption. We also recommend checking centrifugation speed and time, ensuring that tubes are properly balanced. Finally, fresh buffers should be prepared before starting synaptosome preparation.

Problem 2: Variability in proteolytic digestion of postsynaptic proteins.

Possible cause: Partial resistance of postsynaptic proteins to proteolytic degradation due to the densely packed postsynaptic network.

Solution: In the original publication, Boyken et al. [1] observed that certain postsynaptic proteins, such as PSD95 and Homer1, were not degraded, suggesting partial resistance of the postsynaptic density network to proteolytic digestion. In our experience, Homer1 is resistant to trypsin cleavage, while PSD95 is cleaved. However, in the study by Awasthi et al. [17], both PSD95 and Homer1 were cleaved after 10 min of trypsin digestion. This variability may result from differences in synaptosome preparation and/or the antibodies used for western blot detection. We would like to emphasize to readers that although this assay can be used in a high-throughput manner to screen various presynaptic and postsynaptic proteins, their localization should be confirmed with additional methods, such as super-resolution microscopy (e.g., STED), to unequivocally verify proper localization.

Problem 3: Unexpected degradation of proteins during synaptosome preparation.

Possible cause: If protein degradation is observed even in the untreated sample, where trypsin was not added, this suggests degradation due to endogenous cellular proteases released during preparation. Since synaptosome fractionation is performed without protease inhibitors to avoid interference with trypsin activity, we highly recommend conducting all steps at 4 °C and pre-chilling all equipment.

Problem 4: Digestion of presynaptic proteins.

Possible cause: Prolonged digestion time or excessive trypsin digestion.

Solution 1: This protocol relies entirely on the integrity of synaptosomes. If the purified synaptosomes are damaged or broken, trypsin will also access presynaptic proteins. To address this issue, we recommend reducing variability in synaptosome preparations across experiments by monitoring synaptosome yield and purity at intermediate steps (e.g., after each centrifugation). It is especially important to ensure that all materials (e.g., homogenizer) used during synaptosome preparation are detergent-free, as any traces of detergent can compromise synaptosome integrity and thereby affect the final results.

Solution 2: Extended incubation can lead to over-digestion, resulting in degradation of presynaptic proteins. Ensure that trypsin digestion is carefully monitored, carried out at 30 °C for 10 min, followed by immediate centrifugation and the addition of 1 mL sucrose buffer containing 200 μL of 6× loading sample buffer to stop the reaction.

Solution 3: Trypsin potency can vary between different batches. Therefore, it is essential to test each new batch of trypsin before use. Perform a trypsin cleavage assay using various protein-to-protease ratios, such as 50:1, 100:1, or 250:1, to determine the optimal digestion conditions. Select the ratio that provides effective postsynaptic cleavage without leading to over-digestion of presynaptic proteins For example, the old batch of trypsin used in Azarnia Tehran et al. [18] was used with a protein-to-protease ratio of 100:1. In Vollweiter et al. [19], the new batch of trypsin worked best with a protein-to-protease ratio of 50:1 (see Figure 2). With this new batch of trypsin, using a higher trypsin concentration led to cleavage of presynaptic proteins as well (e.g., synaptotagmin1, Syt1).

Problem 5: Weak or absent protein bands in western blot.

Possible cause: Insufficient protein loading.

Solution: We typically load 20–30 μg of protein per well. However, depending on the antibody used, this amount might be insufficient for detection. To address this, optimize the protein loading concentration and adjust the primary and secondary antibody dilutions. Experimenting with different concentrations and dilutions can help enhance the signal and achieve better detection.
